# Ranking Specific Sets of Objects

**DOI:** 10.1007/s13222-017-0264-7

**Published:** 2017-09-11

**Authors:** Jan Maly, Stefan Woltran

**Affiliations:** Institute of Information Systems, TU Wien, Wien, Austria

**Keywords:** Ranking Sets, Complexity

## Abstract

Ranking sets of objects based on an order between the single elements has been thoroughly studied in the literature. In particular, it has been shown that it is in general impossible to find a total ranking – jointly satisfying properties as dominance and independence – on the whole power set of objects. However, in many applications certain elements from the entire power set might not be required and can be neglected in the ranking process. For instance, certain sets might be ruled out due to hard constraints or are not satisfying some background theory. In this paper, we treat the computational problem whether an order on a given subset of the power set of elements satisfying different variants of dominance and independence can be found, given a ranking on the elements. We show that this problem is tractable for partial rankings and NP-complete for total rankings.

## Introduction

The problem of lifting rankings on objects to ranking on sets has been studied from many different view points — see [2] for an excellent survey. Several properties (also called axioms) have been proposed in order to indicate whether the lifted ranking reflects the given order on the elements. Two important axioms are dominance and independence. Roughly speaking, dominance ensures that adding an element which is better (worse) than all elements in a set, makes the augmented set better (worse) than the original one. Independence, on the other hand, states that adding an element $a$ to sets $A$ and $B$ where $A$ is already known to be preferred over $B$, must not make $B\cup\{a\}$ be preferred over $A\cup\{a\}$ (or, in the strict variant, $A\cup\{a\}$ should remain preferred over $B\cup\{a\}$). These axioms were first considered together in the context of decision making under complete uncertainty [9]. There, sets represent the (mutually exclusive) possible outcomes of an action and one tries to rank these sets based on a preference ranking on the outcomes. It is assumed that the probability of each outcome is unknown, i. e., it is only known whether an event is a possible outcome or not. This is a very reductive model. Still, “it does succeed in modelling some empirically interesting situations” [5, p. 2]. Especially, “when the number of possible states of the world is large, an agent of bounded rationality may be incapable of undertaking (or unwilling to undertake) the complex calculations which consideration of the entire rows in the outcome matrix will involve.” [15, p. 2]. Such situations often occur for autonomous agents, for example self driving cars, where “the temporal evolution of situations cannot be predicted without uncertainty because other road users behave stochastically and their goals and plans cannot be measured” [6, p. 1]. Moreover, dominance and independence are also sensible axioms in other contexts, for example bundles of objects of unknown size. Finally, to mention a very different application, Packard [14] used independence and a version of dominance to define plausibility rankings on theories.

However, it is well known that constructing a ranking on the whole power set of objects which jointly satisfies dominance and (strict) independence is, in general, not possible.

### *Example 1 *

Consider the problem of assigning tasks to agents. Let $X=\{t_{1},\dots,t_{n}\}$ be a collection of tasks. Furthermore, assume we know for every agent what tasks they prefer to perform. If there are more tasks than agents, some agents have to perform several tasks, therefore it would be useful to know the preferences over sets of tasks. However, asking for these preferences directly is infeasible even for a reasonable small number of tasks. Therefore, we would like to lift the preferences over tasks to preferences over sets. Furthermore, it seems reasonable that the order on the sets should satisfy dominance and (strict) independence. Unfortunately, for strict independence, this is impossible even for $n=3$. Assume $t_{1}<t_{2}<t_{3}$. Then, $\{t_{1}\}\prec\{t_{1},t_{2}\}$ is implied by dominance, therefore $\{t_{1},t_{3}\}\prec\{t_{1},t_{2},t_{3}\}$ must hold by strict independence. On the other hand, $\{t_{2},t_{3}\}\prec\{t_{3}\}$ is also implied by dominance, therefore, $\{t_{1},t_{2},t_{3}\}\prec\{t_{1},t_{3}\}$ by strict independence. We thus end up with $\{t_{1},t_{2},t_{3}\}\prec\{t_{1},t_{3}\}\prec\{t_{1},t_{2},t_{3}\}$, hence $\prec$ is not an order.

Because of this, other (weaker) axiomatizations were proposed (see for example [7], or more recently, [4] and [10] among many others). However, in many applications one does not need to order the entire power set (for example, some tasks cannot be performed in parallel). In these cases, it may be possible to construct rankings that jointly satisfy dominance and (strict) independence.

### *Example 2 *

Let $X$ be as above. Now assume $\{t_{1},t_{2},t_{3}\}$ is not a possible combination of tasks, for example, because fulfilling all three tasks at once is not feasible. Then, for example

$$\{t_{1}\}\prec\{t_{1},t_{2}\}\prec\{t_{2}\}\prec\{t_{1},t_{3}\}\prec\{t_{2},t_{3}\}\prec\{t_{3}\}$$

is a total order that satisfies dominance and strict independence (respecting the underlying linear order $t_{1}<t_{2}<t_{3}$).

In this paper, we investigate exactly this situation, i. e., lifting rankings to specific sets of elements. In the literature, this scenario seems to be rather neglected, so far. The only exception we are aware of deals with subsets of a fixed cardinality [3]. In particular, we are interested in the complexity of computing, if possible, rankings on arbitrary subsets of the power set that satisfy dominance and (strict) independence. To do so, we first give a new definition for dominance which appears more suitable in such a setting (for more details, see Section 3). Then, we consider the following problem: Given a ranking on elements, and a set $S$ of sets of elements, does there exist a strict (partial) order on $S$ that satisfies $D$ and $I$ (where $D$ is either standard dominance or our notion of dominance and $I$ is independence or strict independence)? We show that the problem is either trivial or easy to solve for the case of partial orders. Our main result is NP-completeness for the case when total orders are required.

The remainder of the paper is organized as follows. In the next section, we recall some basic concepts. In Section 3 we discuss why standard dominance can be seen as too weak in our setting and propose an alternative definition. Section 4 contains our main results. We conclude the paper in Section 5 with a summary and pointers to future work.

This paper is an extended version of [11].

## Background

The formal framework we want to consider in the following consists of a finite^1^ nonempty set $X$, equipped with a linear order $<$ and a subset $\mathcal{X}\subseteq\mathcal{P}(X) \backslash \{\emptyset\}$ of the power set of $X$ not containing the empty set. We want to find a binary relation $\prec$ on $\mathcal{X}$ that satisfies some niceness conditions. We will consider several kinds of relations. We recall the relevant definitions.

### Definition 1 

A binary relation is called a strict partial order, if it is irreflexive and transitive. A strict or linear order is a total strict partial order. A binary relation is called a preorder, if it is reflexive and transitive. A (weak) order is a total preorder. If $\preceq$ is a weak or a preorder on a set $X$, for all $x,y\in X$, the corresponding strict order $\prec$ is defined by $x\prec y$ if $x\preceq y$ and $y\not\preceq x$ hold.

Additionally, we need the following notions:

### Definition 2 

For a pre- or weak order $\preceq$, we write $x\sim y$ if $x\preceq y$ and $y\preceq x$ hold. Let $A\in\mathcal{X}$ be a set of elements of $X$. Then we write $\max(A)$ for the maximal element of $A$ with respect to $<$ and $\min(A)$ for the minimal element of $A$ with respect to $<$. Furthermore, we say a relation $R$ on a set $\mathcal{X}$ extends a relation $S$ on $\mathcal{X}$ if $xSy$ implies $xRy$ for all $x,y\in\mathcal{X}$. Finally, we say a relation $R$ on $\mathcal{X}$ is the transitive closure of a relation $S$ on $\mathcal{X}$ if the existence of a sequence $x_{1}Sx_{2}S\dots Sx_{k}$ implies $x_{1}Rx_{k}$ for all $x_{1},x_{k}\in\mathcal{X}$ and $R$ is the smallest relation with this property. We write $\mathit{trcl}(S)$ for the transitive closure of $S$.

Many different axioms a good order should satisfy are discussed in the literature (an overview over the relevant interpretations and the corresponding axioms can be found in the survey [2]). The following axioms “have very plausible intuitive interpretations” [2, p. 11] for decision making under complete uncertainty and belong to the most extensively studied ones. (We added conditions of the form $X\in\mathcal{X}$ that are not necessary if $\mathcal{X}=\mathcal{P}(X)\backslash\{\emptyset\}$ holds.)

### Axiom 1 (Extension Rule)

For all $x,y\in X$, such that $\{x\},\{y\}\in\mathcal{X}$:

$$x<y\text{ implies }\{x\}\prec\{y\}.$$

### Axiom 2 (Dominance)

For all $A\in\mathcal{X}$ and all $x\in X$, such that $A\cup\{x\}\in\mathcal{X}$:

$$\begin{aligned} & y<x\text{ for all }y\in A\text{ implies }A\prec A\cup\{x\};\\ & x<y\text{ for all }y\in A\text{ implies }A\cup\{x\}\prec A.\end{aligned}$$

### Axiom 3 (Independence)

For all $A,B\in\mathcal{X}$ and for all $x\in X\backslash(A\cup B)$, such that $A\cup\{x\},B\cup\{x\}\in\mathcal{X}$:

$$A\prec B\text{ implies }A\cup\{x\}\preceq B\cup\{x\}.$$

### Axiom 4 (Strict Independence)

For all $A,B\in\mathcal{X}$ and for all $x\in X\backslash(A\cup B)$, such that $A\cup\{x\},B\cup\{x\}\in\mathcal{X}$:

$$A\prec B\text{ implies }A\cup\{x\}\prec B\cup\{x\}.$$

### *Example 3 *

Take $X=\{1,2,3,4\}$ with the usual linear order and

$$\mathcal{X}=\{\{3\},\{4\},\{1,3\},\{2,3\},\{1,4\},\{1,2,3\},\{1,3,4\}\}.$$

Then the extension rule implies $\{3\}\prec\{4\}$, dominance implies $\{1,3\}\prec\{1,3,4\}$, $\{1,2,3\}\prec\{2,3\}\prec\{3\}$ and $\{1,4\}\prec\{4\}$ but not $\{3\}\prec\{4\}$. Furthermore, (strict) independence lifts the preference between $\{2,3\}$ and $\{3\}$ to $\{1,2,3\}$ and $\{1,3\}$, i. e., in combination with dominance, independence implies $\{1,2,3\}\preceq\{1,3\}$ and strict independence implies $\{1,2,3\}\prec\{1,3\}$.

Every reasonable order should satisfy the extension rule. If we assume $\mathcal{X}=\mathcal{P}(X)\backslash\{\emptyset\}$, the extension rule is implied by dominance [2]. Therefore, a natural task is to find a total order on $\mathcal{P}(X)\backslash\{\emptyset\}$ that satisfies dominance together with (some version of) independence. However, in their seminal paper [9], Kannai and Peleg have shown that this is impossible for regular independence and dominance if $|X|\geq 6$ and $\mathcal{X}=\mathcal{P}(X)\backslash\{\emptyset\}$ hold. Barberà and Pattanaik [1] showed that for strict independence and dominance this is impossible even for $|X|\geq 3$ and $\mathcal{X}=\mathcal{P}(X)\backslash\{\emptyset\}$ (see Example 1 for a proof of the statement).

If we abandon the condition $\mathcal{X}=\mathcal{P}(X)\backslash\{\emptyset\}$, the situation is not as clear. As we have seen in Example 2 there are sets $\mathcal{X}\subseteq\mathcal{P}(X)\backslash\{\emptyset\}$ with $|X|\geq 3$ such that there is an order on $\mathcal{X}$ satisfying strict independence and dominance.

## A Stronger Form of Dominance

Many results regularly used in the setting of $\mathcal{X}=\mathcal{P}(X)\backslash\{\emptyset\}$ are not true in the more general case. For example, in contrast to the result stated above, the extension rule is not implied by dominance as we have seen in Example 3. Furthermore, it could be argued that $\{1,3\}\prec\{1,4\}$ should hold in that example which would be implied by dominance and independence if $\{3,4\}\in\mathcal{X}$ would hold, because $\{3,4\}\prec\{4\}$ holds by dominance and so $\{1,3,4\}\preceq\{1,4\}$ by independence. Hence, $\{1,3\}\prec\{1,3,4\}\preceq\{1,4\}$ implies $\{1,3\}\prec\{1,4\}$ by transitivity.

Furthermore, for the set $X$ from Example 3, dominance does not even imply $\{1\}\prec\{1,2,3\}$ if $\{1,2\}$ is not in the family. Therefore, it is reasonable to ask for a stronger version of dominance that behaves nicely in the general case. We observe that $x<y$ for all $y\in A$ implies $\max(A\cup\{x\})=\max(A)$ and $\min(A\cup\{x\})<\min(A)$; whereas $y<x$ for all $y\in A$ implies $\max(A)<\max(A\cup\{x\})$ and $\min(A\cup\{x\})=\min(A)$. We claim that every dominance-like axiom should satisfy this property. Therefore, we can use this property to define a “maximal” version of dominance which can be seen as a special case of Pareto dominance [12].

### Axiom 5 (Maximal Dominance)

For all $$A,B\in\mathcal{X}$$,

$$\begin{aligned} & \left(\max(A)\leq\max(B)\land\min(A)<\min(B)\right)\text{ or }\\ & \left(\max(A)<\max(B)\land\min(A)\leq\min(B)\right)\text{ implies }A\prec B.\end{aligned}$$

This axiom trivially implies the extension rule and of course dominance. Looking again at the family introduced in Example 3 maximal dominance implies all preferences implied by either dominance or by the extension rule and additionally $\{1,3\}\prec\{1,4\}$. Furthermore, if $\mathcal{X}$ is sufficiently large or even $\mathcal{X}=\mathcal{P}(X)\backslash\{\emptyset\}$, dominance and independence imply maximal dominance.

### Proposition 1 

*Let* $\mathcal{X}=\mathcal{P}(X)\backslash\{\emptyset\}$ *. Then every transitive relation that satisfies dominance and independence also satisfies maximal dominance and independence.*

### *Proof *

Let $\preceq$ be a transitive relation that satisfies dominance and independence. We show that $\preceq$ satisfies maximal dominance using the following observation due to Kannai and Peleg in [9]:

**Observation** $$A\sim\{\min(A),\max(A)\}$$.

We can assume w.l.o.g. that $A$ has more than two elements. We enumerate $A$ by $A=\{a_{1},a_{2},\dots,a_{k}\}$ such that $a_{i}<a_{j}$ holds for all $i<j$. Using transitivity and dominance, it is easy to see that $\{a_{1}\}\prec\{a_{1},a_{2},\dots,a_{k-1}\}$ holds. This implies, by independence, $\{a_{1},a_{k}\}\preceq A$. Analogously, we get $\{a_{2},a_{2},\dots,a_{k}\}\prec\{a_{k}\}$ and $A\preceq\{a_{1},a_{k}\}$ and therefore $A\sim\{a_{1},a_{k}\}=\{\min(A),\max(A)\}$.◊

Using this observation we can prove that $\max(A)=\max(B)$ and $\min(A)<\min(B)$ implies $A\prec B$ by the following argument:

$$\begin{aligned} A & \sim\{\min(A),\max(A)\}\sim\{\min(A),\min(B),\max(A)\}\\ & \prec\{\min(B),\max(A)\}=\{\min(B),\max(B)\}\sim B.\end{aligned}$$

The other case is proven analogously, hence $\preceq$ satisfies maximal dominance.□

It would be possible to define several other versions of dominance of intermediate strength. We will only consider dominance and maximal dominance. As we will see our results justify this approach; in particular, since both versions yield equal complexity results.

## Main Results

We studied 8 problems in total, as defined below.^2^ Our results are summarized in Table 1.

### Problem 1 (The Partial (Maximal) Dominance Strict Independence problem)

Given a linearly ordered set $X$ and a set $\mathcal{X}\subseteq\mathcal{P}(X)\backslash\{\emptyset\}$, decide if there is a partial order $\prec$ on $\mathcal{X}$ satisfying (maximal) dominance and strict independence.

### Problem 2 (The Partial (Maximal) Dominance Independence problem)

Given a linearly ordered set $X$ and a set $\mathcal{X}\subseteq\mathcal{P}(X)\backslash\{\emptyset\}$, decide if there is a preorder $\preceq$ on $\mathcal{X}$ satisfying (maximal) dominance and independence.

### Problem 3 (The (Maximal) Dominance Strict Independence problem)

Given a linearly ordered set $X$ and a set $\mathcal{X}\subseteq\mathcal{P}(X)\backslash\{\emptyset\}$, decide if there is a strict total order $\prec$ on $\mathcal{X}$ satisfying (maximal) dominance and strict independence.

### Problem 4 (The (Maximal) Dominance Independence problem)

Given a linearly ordered set $X$ and a set $\mathcal{X}\subseteq\mathcal{P}(X)\backslash\{\emptyset\}$, decide if there is a total order $\preceq$ on $\mathcal{X}$ satisfying (maximal) dominance and independence.

**Table 1:** Main results: Complexity of deciding Problems 1–4

	Not total	Total
Dominance + Independence	always yes^a^	NP-comp.
Max. Dominance + Independence	always yes^a^	NP-comp.
Dominance + Strict Ind.	in P	NP-comp.
Max. Dominance + Strict Ind.	in P	NP-comp.

^a^ Constructing the order can be done in polynomial time

### Partial Orders

First, we consider the Partial (Maximal) Dominance Independence problem. We can define a preorder that satisfies independence and maximal dominance (and therefore also dominance) on all $\mathcal{X}$.

#### Definition 3 

Given a set $X$, a linear order $<$ on $X$ and a family $\mathcal{X}\subseteq\mathcal{P}(X)\backslash\emptyset$, we define a relation $\preceq_{m}$ as $A\preceq_{m}B$ iff $\max(A)\leq\max(B)$ and $\min(A)\leq\min(B)$.

Observe that it is obviously possible, given $X$, $<$ and $\mathcal{X}$, to construct $\preceq_{m}$ in polynomial time.

#### Theorem 4.1

*For every linearly ordered* $X$ *and every family* $\mathcal{X}\subseteq\mathcal{P}(X)\backslash\emptyset$ *,* $\preceq_{m}$ *is a preorder and satisfies maximal dominance and independence.*

#### *Proof*

Obviously, $\preceq_{m}$ is reflexive and transitive, because $\leq$ is reflexive and transitive. Furthermore, the corresponding strict order $\prec_{m}$ satisfies maximal dominance. Assume, w.l.o.g., $\min(A)<\min(B)$ and $\max(A)\leq\max(B)$. Then $A\preceq_{m}B$ by definition and $B\not\preceq_{m}A$ because $\min(B)\not\leq\min(A)$, so $A\prec_{m}B$. Finally, assume $A\prec_{m}B$ and $A\cup\{x\},B\cup\{x\}\in\mathcal{X}$ for $x\not\in A\cup B$ and, w.l.o.g., $\min(A)<\min(B)$ and $\max(A)\leq\max(B)$. If $\min(A)<x$ we know $\min(A\cup\{x\})<\min(B\cup\{x\})$ and $\max(A\cup\{x\})\leq\max(B\cup\{x\})$; if $x<\min(A)$ we get $\min(A\cup\{x\})\leq\min(B\cup\{x\})$, and $\max(A\cup\{x\})\leq\max(B\cup\{x\})$. Hence, $A\cup\{x\}\preceq_{m}B\cup\{x\}$.□

#### *Example 4 *

Consider once again the family from Example 3. $\preceq_{m}$ consists of the following preferences on that family:

$$\begin{aligned} \{1,3\} & \sim_{m}\{1,2,3\}\prec_{m}\{1,3,4\}\sim_{m}\{1,4\}\prec_{m}\{4\}, \\ & \{1,3\}\sim_{m}\{1,2,3\}\prec_{m}\{2,3\}\prec_{m}\{3\}\prec_{m}\{4\}. \end{aligned}$$

Next, we consider the Partial Dominance Strict Independence problem. As we have seen in Example 1 and 2, only some sets $\mathcal{X}$ allow such an order. In order to decide if a set admits a partial order we build a minimal transitive relation satisfying dominance and strict independence. First, we build a minimal transitive relation satisfying dominance. It is worth noting that a very similar relation can be defined for maximal dominance. With this relation, all results in this section can be proven for maximal dominance the same way.

#### Definition 4 

Given a set $X$, a linear order $<$ on $X$ and a family $\mathcal{X}\subseteq\mathcal{P}(X)\backslash\emptyset$, we define a relation $\prec_{d}$ on $\mathcal{X}$ in the following way: If $A,A\cup\{x\}\in\mathcal{X}$, then

1. $A\prec_{d}A\cup\{x\}$ if $y<x$ for all $y\in A$.
2. $A\cup\{x\}\prec_{d}A$ if $x<y$ for all $y\in A$.

We define the relation $\prec_{d}^{t}$ on $\mathcal{X}$ by $\prec_{d}^{t}:=\mathit{trcl}(\prec_{d})$.

This relation has the following useful property.

#### Proposition 2 

*For every linearly ordered set* $X$ *and every family* $\mathcal{X}\subseteq\mathcal{P}(X)\backslash\emptyset$ *,* $\prec_{d}^{t}$ *is a partial order and a partial order on* $\mathcal{X}$ *satisfies dominance if and only if it extends* $\prec_{d}^{t}$ *.*

#### *Proof *

Obviously, $\prec_{d}^{t}$ is transitive. Furthermore, $\prec_{d}^{t}$ is irreflexive as $A\prec_{d}^{t}B$ implies $\max(A)<\max(B)$ or $\min(A)<\min(B)$ and $<$ is irreflexive.

By definition, a relation satisfies dominance if and only if it extends $\prec_{d}$ and a transitive relation extending $\prec_{d}$ also extends $\prec_{d}^{t}$ by the minimality of $\mathit{trcl}$.□

We want to extend this relation to a minimal relation for strict independence and dominance.

#### Definition 5 

Given a set $X$, a linear order $<$ on $X$ and a family $\mathcal{X}\subseteq\mathcal{P}(X)\backslash\emptyset$, we build a relation $\prec_{\infty}$ on $\mathcal{X}$ by induction. First, we set $\prec_{0}^{t}:=\prec_{d}^{t}$. Now let $\prec_{n}^{t}$ be defined. For $\prec_{n+1}$ we select sets $A,B,A\backslash\{x\},B\backslash\{x\}\in\mathcal{X}$ with $x\in X$, $A\backslash\{x\}\prec_{n}^{t}B\backslash\{x\}$ but not $A\prec^{n}_{t}B$ and set $C\prec_{n+1}D$ if $C\prec_{n}^{t}D$ or $C=A$ and $D=B$ holds. Then, we set $\prec_{n+1}^{t}:=\mathit{trcl}(\prec_{n+1})$. In the end, we set $\prec_{\infty}=\bigcup_{n}\prec^{t}_{n}$.

#### *Example 5 *

Consider the family from Example 3, i. e.,

$$\mathcal{X}=\{\{3\},\{4\},\{1,3\},\{2,3\},\{1,4\},\{1,2,3\},\{1,3,4\}\}.$$

Then, $\prec_{\infty}$ consists of the following preferences:

$$\begin{aligned} & \{1,3\}\prec_{\infty}\{1,3,4\},\\ & \{1,2,3\}\prec_{\infty}\{2,3\}\prec_{\infty}\{3\},\\ & \{1,4\}\prec_{\infty}\{4\},\\ & \{1,2,3\}\prec_{\infty}\{1,3\}.\end{aligned}$$

In order to prove that this is actually a minimal order for dominance and strict independence, we have to introduce another concept we call links.

#### Definition 6 

A $\prec_{\infty}$-link from $A$ to $B$ in $\mathcal{X}$ is a sequence $A=:C_{0},C_{1},\dots,C_{n}:=B$ with $C_{i}\in\mathcal{X}$ for all $i\leq n$ such that, for all $i<n$, either $C_{i}\prec_{d}C_{i+1}$ holds or there is a link between $C_{i}\backslash\{x\}$ and $C_{i+1}\backslash\{x\}$ for some $x\in X$.

We show that $\prec_{\infty}$-links indeed characterize $\prec_{\infty}$.

#### Lemma 1 

*For* $A,B\in\mathcal{X}$ *,* $A\prec_{\infty}B$ *implies that there is a* $\prec_{\infty}$ *-link from* $A$ *to* $B$ *and if there is a* $\prec_{\infty}$ *-link from* $A$ *to* $B$ *then* $A\prec^{*}B$ *holds for every transitive relation* $\prec^{*}$ *that satisfies dominance and strict independence.*

In order to prove this result, we need another definition.

#### Definition 7 

For every pair $A\prec_{\infty}B$, there is a minimal $k$ such that $A\prec_{k}^{t}B$ holds. We call this the $\prec_{\infty}$-rank of the pair.

Furthermore, we define the $\text{rank}(C_{1},C_{2},\dots,C_{n})$ of a $\prec_{\infty}$-link $C_{1},C_{2},\dots,C_{n}$ from $C_{1}$ to $C_{n}$:

- $$\text{rank}^{*}(C_{i},C_{i+1})=0$$ if $$C_{i}\prec_{d}C_{i+1}$$,
- $$\text{rank}^{*}(C_{i},C_{i+1})=\text{rank}(C_{i}\backslash\{x\},C_{i+1}\backslash\{x\})$$,
- $$\text{rank}(C_{1},C_{2},\dots,C_{n})=\max\{\text{rank}^{*}(C_{i},C_{i+1})\mid i<n\}+1$$.

Now we can prove Lemma 1:

#### *Proof *

Assume $A\prec_{\infty}B$. We prove that a $\prec_{\infty}$-link exists by induction on the $\prec_{\infty}$-rank of $A,B$. If $A\prec_{d}^{t}B$, then there is sequence $A=C_{1},C_{2},\dots,C_{n}=B$ such that $C_{i}\prec_{d}C_{i+1}$ holds for all $i<n$, hence there is a $\prec_{\infty}$-link from $A$ to $B$. Now assume $A,B$ has $\prec_{\infty}$-rank $k$ and for every pair with $\prec_{\infty}$-rank $k-1$ or less there is a $\prec_{\infty}$-link from $C$ to $D$. There is a sequence $A=C_{0}\prec_{k}C_{1}\dots C_{n-1}\prec_{k}C_{n}=B$. For every $i<n$ either $C_{i}\prec_{d}C_{i+1}$ or $C_{i}\backslash\{y\}\prec_{k-1}^{t}C_{i+1}\backslash\{y\}$ holds, which implies by induction that there is a $\prec_{\infty}$-link from $C_{i}\backslash\{y\}$ to $C_{i+1}\backslash\{y\}$. Hence there is a $\prec_{\infty}$-link from $A$ to $B$.

Now, let $\prec$ be a transitive relation that satisfies dominance and strict independence and assume there is a $\prec_{\infty}$-link $A=C_{1},C_{2},\dots,C_{n}=B$ from $A$ to $B$. We prove $A\prec B$ by induction on the rank of the $\prec_{\infty}$-link. First, assume $\text{rank}(C_{1},C_{2},\dots,C_{n})=1$, then $C_{i}\prec_{d}C_{i+1}$ holds for all $i<n$, hence $A\prec B$ holds by dominance and transitivity. Now assume $\text{rank}(C_{1},C_{2},\dots,C_{n})=k$ and for all $\prec_{\infty}$-links with $\text{rank}(C^{*}_{1},C^{*}_{2},\dots,C^{*}_{n})<k$ we know $C_{1}^{*}\prec C_{n}^{*}$. By induction, for every $i<n$ either $C_{i}\prec_{d}C_{i+1}$ or $C_{i}\backslash\{x\}\prec C_{i+1}\backslash\{x\}$ holds. This implies that $C_{i}\prec C_{i+1}$ holds for all $i<n$, because $\prec$ satisfies dominance and strict independence. Therefore $A\prec B$ by transitivity.□

Using this lemma, we can show now that $\prec_{\infty}$ is indeed a minimal relation for dominance and strict independence.

#### Theorem 4.2

*Given a set* $X$ *, a linear order* $<$ *on* $X$ *and a family* $\mathcal{X}\subseteq\mathcal{P}(X)\backslash\{\emptyset\}$ *, there is a partial order on* $\mathcal{X}$ *that satisfies dominance and strict independence if and only if* $\prec_{\infty}$ *is irreflexive on* $\mathcal{X}$ *.*

#### *Proof *

$\prec_{\infty}$ satisfies dominance as it extends $\prec_{d}^{t}$. By construction it also satisfies strict independence and transitivity: $A_{1}\prec_{\infty}A_{2}\prec_{\infty}\dots\prec_{\infty}A_{k}$ implies $A_{1}\prec^{t}_{n}A_{2}\prec^{t}_{n}\dots\prec^{t}_{n}A_{k}$ for some $n\in N$ but then $A_{1}\prec_{n}^{t}A_{k}$ holds by the transitivity of $\prec_{n}^{t}$ and therefore $A_{1}\prec_{\infty}A_{k}$. Now assume $A\prec_{\infty}B$ and hence $A\prec^{t}_{n}B$ for some $n$ and $A\cup\{x\}\not\prec_{n}^{t}B\cup\{x\}\in\mathcal{X}$ for some $x\not\in A\cup B$. Then $A,B,A\cup\{x\},B\cup\{x\}$ is picked for some $l$ with $n<l$ and $A\cup\{x\}\prec_{l}B\cup\{x\}$ is set, hence $A\cup\{x\}\prec_{\infty}B\cup\{x\}$. Therefore, if $\prec_{\infty}$ is irreflexive, it is a partial order satisfying dominance and strict independence.

On the other hand, if $\prec_{\infty}$ is not irreflexive no strict partial order can extend it. But every strict partial order on $\mathcal{X}$ satisfying dominance and strict independence must be an extension of $\prec_{\infty}$. Assume otherwise there is a strict partial order $\prec$ on $\mathcal{X}$ satisfying dominance and strict independence that does not extend $\prec_{\infty}$, i. e., there are sets $A,B\in\mathcal{X}$ such that $A\prec_{\infty}B$ holds but not $A\prec B$. By Lemma 1 there is a $\prec_{\infty}$-link from $A$ to $B$. This implies, by Lemma 1, $A\prec B$ because $\prec$ is transitive and satisfies dominance and strict independence. Contradiction. Therefore no partial order on $\mathcal{X}$ can satisfy dominance and strict independence, if $\prec_{\infty}$ is irreflexive.□

Using this result, we can define a polynomial time algorithm for the Partial Dominance Strict Independence Problem.

#### Corollary 1 

*The Partial Dominance Strict Independence problem is in* $P$ *.*

#### *Proof *

Computing $\prec_{\infty}$ can obviously be done in polynomial time because the construction always stops after at most $|n\times n|=n^{2}$ steps. Then checking if $\prec_{\infty}$ is irreflexive only requires checking if $A\prec_{\infty}A$ holds for some $A$.□

Finally, links give us an easy characterization of sets $\mathcal{X}$ for which $\prec_{\infty}$ is irreflexive.

#### Corollary 2 

$\prec_{\infty}$ *is irreflexive if and only if there is no set* $A\in\mathcal{X}$ *such that there is a* $\prec_{\infty}$ *-link from* $A$ *to* $A$ *.*

#### *Proof *

$\prec_{\infty}$ is transitive and satisfies dominance and strict independence, hence Lemma 1 tells us, that $A\prec_{\infty}A$ if and only if there is a $\prec_{\infty}$ link from A to A.

### Total Orders

We show that it is, in general, not possible to construct a (strict) total order satisfying both (maximal) dominance and (strict) independence deterministically in polynomial time^3^. We do this by a reduction from betweenness.

#### Problem 5 (Betweenness)

Given a set $V=\{v_{1},v_{2},\dots,v_{n}\}$ and a set of triples $R\subseteq V^{3}$, does there exist a strict total order on $V$ such that $a<b<c$ or $a> b> c$ holds for all $(a,b,c)\in R$.

Betweenness is known to be NP-hard [13]. We use this result to show NP-hardness for all four versions of the (Maximal) Dominance (Strict) Independence problem. The idea is, roughly, to represent the elements of $V$ by sets which are not directly comparable via the axioms of dominance or independence. Hence, in order to find a total order, we need to guess how these sets are ordered. Starting from this guess we need to “maximize” this initial order in such a way that for each triple $(a,b,c)$ both $a<b> c$ and $a> b<c$ would lead to a circle in every order satisfying dominance and independence. However, this requires a number of carefully chosen additional sets as we will detail below.

#### Theorem 4.3

*The Maximal Dominance Strict Independence problem, the Dominance Strict Independence problem, the Maximal Dominance Independence problem and the Dominance Independence problem are NP-complete.*

It is clear that all four problems are in NP. We can guess a binary relation and then check if it has all properties we want. It is well known that checking for transitivity and (ir-) reflexivity can be done in polynomial time. Checking (maximal) dominance only requires an easy check for every pair of sets and (strict) independence an equally easy check for every quadruple of sets. It is clear that this can be done in polynomial time. In what follows, we split the proof of the NP-hardness in four parts, one for each problem.

#### The Maximal Dominance Strict Independence problem

##### *Proof *

Let $(V,R)$ be an instance of betweenness with $V=\{v_{1},v_{2},\ldots,v_{n}\}$. We construct an instance $(X,<,\mathcal{X})$ of the Maximal Dominance Strict Independence problem. We set $X=\{1,2,\dots,N\}$ equipped with the usual linear order, for $N=8n^{3}+2n+2$. Then, we construct the family $\mathcal{X}$ stepwise. The family contains for every $v_{i}\in V$ a set $V_{i}$ of the following form (see Figure 3):

$$V_{i}:=\{1,N\}\cup\{i+1,i+2,\dots,N-i\}.$$

**Fig. 1:**
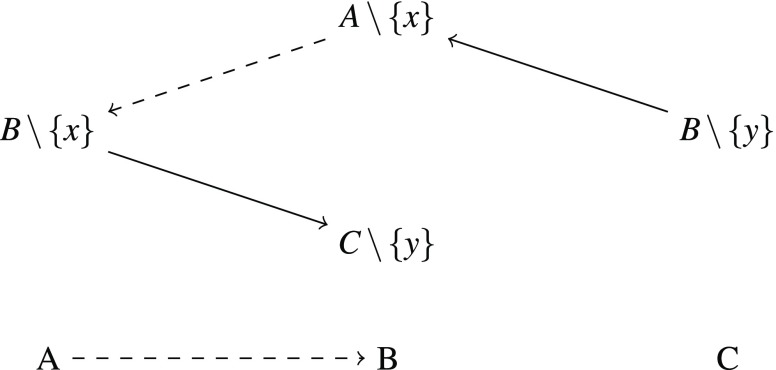
Family that forces that $A\prec B$ leads to $B\prec C$

**Fig. 2:**
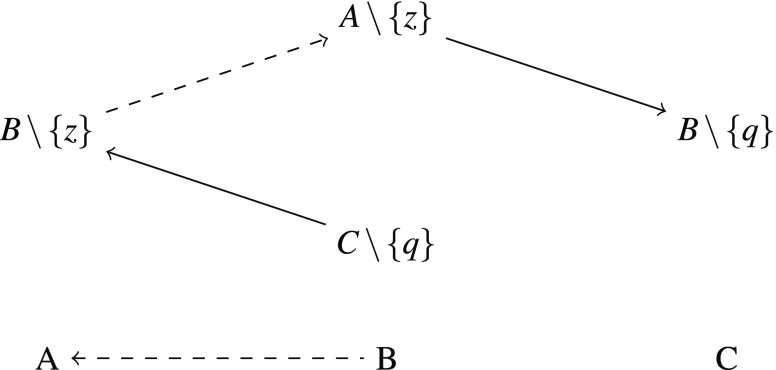
Family that forces that $A\succ B$ leads to $B\succ C$

Furthermore, for every triple from $R$ we want to enforce $A\prec B\prec C$ or $A\succ B\succ C$ by adding two families of sets as shown in Figure 1 and Figure 2 with $q,x,y,z\in X$. The solid arrows represent preferences that are forced through maximal dominance and strict independence. The family in Figure 1 makes sure that every total strict order satisfying independence that contains $A\prec B$ must also contain $B\prec C$. Similarly, the family in Figure 2 makes sure that $A\succ B$ leads to $B\succ C$.

We implement this idea for all triples inductively. For every $1\leq i\leq|R|$, pick a triple $(v_{l},v_{j},v_{m})\in R$ and set $k=n+1+8i$. Let $(A,B,C)=(V_{l},V_{j},V_{m})$ be the triple of sets coding the triple of elements $(v_{l},v_{j},v_{m})$. We add the following sets:

$$\begin{aligned} & A\backslash\{k\},B\backslash\{k\},B\backslash\{k+1\},C\backslash\{k+1\}, \\ & A\backslash\{k+2\},B\backslash\{k+2\},B\backslash\{k+3\},C\backslash\{k+3\}.\end{aligned}$$

These sets correspond to the sets $A\backslash\{x\},B\backslash\{x\},\dots,C\backslash\{q\}$ in Figure 1 and Figure 2. Observe that the inductive construction guarantees that every constructed set is unique. We now have to force the preferences

$$\begin{aligned} & B\backslash\{k+1\}\prec A\backslash\{k\},\quad B\backslash\{k\}\prec C\backslash\{k+1\}, \\ & A\backslash\{k+2\}\prec B\backslash\{k+3\},\quad C\backslash\{k+3\}\prec B\backslash\{k+2\}.\end{aligned}$$

For technical reasons^4^, we add sets $A\backslash\{k,k+4\},B\backslash\{k+1,k+4\}$. Then, observe that, by construction, either $B\backslash\{1,k+1,k+4\}\prec A\backslash\{1,k,k+4\}$ or $B\backslash\{k+1,k+4,N\}\prec A\backslash\{k,k+4,N\}$ is implied by maximal dominance. We add $A\backslash\{1,k,k+4\}$ and $B\backslash\{1,k+1,k+4\}$ in the first case and $A\backslash\{k,k+4,N\}$ and $B\backslash\{k+1,k+4,N\}$ in the second case (see Figure 4). This ensures $B\backslash\{k+1\}\prec A\backslash\{k\}$ by strict independence. In the same way, we can force the other preferences using $k+5,k+6$ and $k+7$ instead of $k+4$.

We repeat this with a new triple $(v_{i}^{\prime},v_{j}^{\prime},v_{m}^{\prime})\in R$ until we treated all triples in $R$. Observe that there are at most $n^{3}$ triples, thus, for every triple, the values $k,\dots,k+7$ lie between $n+1$ and $N-n$, hence are element of every $V_{i}$. In total, we add $24$ sets per triple. Therefore, $\mathcal{X}$ contains $n+24n^{3}$ sets.

It is easy to see, that, by construction, for every strict total order on $\mathcal{X}$ satisfying maximal dominance and strict independence, we have

$$\begin{aligned} & A\backslash\{k\}\prec B\backslash\{k+1\},\quad C\backslash\{k+1\}\prec B\backslash\{k\}, \\ & B\backslash\{k+3\}\prec A\backslash\{k+2\},\quad B\backslash\{k+2\}\prec C\backslash\{k+3\}.\end{aligned}$$

Now assume there is a strict total order on $\mathcal{X}$ satisfying maximal dominance and strict independence. We claim that the relation defined by $v_{i}<v_{j}$ iff $V_{i}\prec V_{j}$ is a positive witness for $(V,R)$. By definition this is a strict total order. So assume there is a triple $(a,b,c)$ such that $a> b<c$ or $a<b> c$ holds. We treat the first case in detail: $a> b<c$ implies $A\succ B\prec C$. This implies by the strictness of $\prec$ and strict dominance $A\backslash\{k\}\succ B\backslash\{k\}$ and $B\backslash\{k+1\}\prec C\backslash\{k+1\}$. However, then

$$\begin{aligned} & A\backslash\{k\}\succ B\backslash\{k\}\succ C\backslash\{k+1\} \\ & \succ B\backslash\{k+1\}\succ A\backslash\{k\}\end{aligned}$$

contradicts the assumption that $\prec$ is transitive and irreflexive. Similarly, the second case leads to a contradiction.

Now assume that there is a strict total order on $V$ satisfying the restrictions from $R$. We use this to construct an order on $\mathcal{X}$. We set $V_{i}\prec V_{j}$ iff $v_{i}<v_{j}$ holds. Furthermore, we set $A\prec B$ for all $A,B\in\mathcal{X}$ if it is implied by dominance. Then, we apply strict independence twice and once “reverse” strict independence^5^, i. e., $A\prec B$ implies $A\backslash\{x\}\prec B\backslash\{x\}$ for $A,B,A\backslash\{x\},B\backslash\{x\}\in\mathcal{X}$. We claim that all possible instances of strict independence are decided already by this order. If $A=V_{i}$ for $i\leq n$, then there is no set $A\cup\{x\}$ in $\mathcal{X}$. If $A=V_{i}\backslash\{x\}$ for some $i\leq n$ and $x\in X$, then $x$ is the only element of $X$ such that $A\cup\{x\}\in\mathcal{X}$ holds. But then there can only be one other set $B$ with $B\cup\{x\}\in\mathcal{X}$ and $B=V_{j}\backslash\{x\}$ hence a preference between $A$ and $B$ was already introduced by reverse strict independence. Analogously in the cases $A=V_{i}\backslash\{x,y\}$ and $A=V_{i}\backslash\{x,y,z\}$ for $i\leq n$ and $x\neq y\neq z\in X$, every possible instance of strict independence is already decided by dominance and two applications of strict independence.

It can easily be seen, that this construction does not lead to circles, if we start with a positive instance of betweenness: Every set of the form $A=V_{i}\backslash\{x,y,z\}$ is only comparable to other sets by maximal dominance. Every set of the form $A=V_{i}\backslash\{x,y\}$ is only comparable by maximal dominance or to another set of the same form. The order on sets of this form mirrors the order on sets of the form $A=V_{i}\backslash\{x,y,z\}$ which is produced by maximal dominance and hence is circle free. Finally sets of the form $A=V_{i}\backslash\{x\}$ or $A=V_{i}$ are only comparable to other sets by maximal dominance or if this is intended by the construction. Hence, the order on these sets is circle free, if we started with a positive instance of betweenness.

Finally, we can extend this order to a total order because extensions do not produce new instances of strict independence.□

**Fig. 3:**
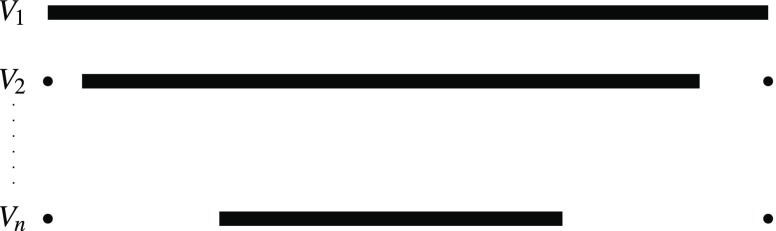
Sketch of the sets $V_{1},V_{2}$ and $V_{n}$

**Fig. 4:**
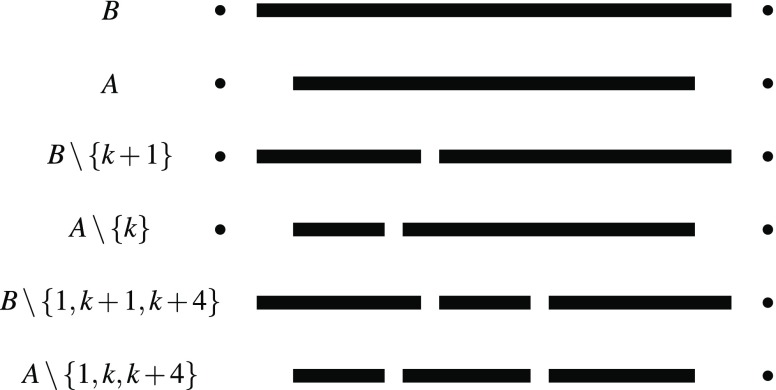
Sketch of the sets $A,B,A\backslash\{k\},B\backslash\{k+1\},A\backslash\{1,k,k+4\}$ and $B\backslash\{1,k+1,k+4\}$

#### The Dominance Strict Independence problem

##### *Proof *

We construct an instance $(X,<,\mathcal{X})$ of the Dominance Strict Independence problem in a similar fashion as above. We take the same $X$ and $<$ and add the same sets to $\mathcal{X}$. In order to make the reduction work for the Dominance Strict Independence problem, we have to add more sets.

Observe that maximal dominance is only needed in the reduction for the Maximal Dominance Strict Independence problem to introduce preferences like $A\backslash\{1,k,k+4\}\prec B\backslash\{1,k+1,k+4\}$. We can enforce these preferences also using strict independence and regular dominance using a construction as in the proof of Proposition 1.

For every $k$ used in the reduction, let $(A,B,C)$ be the triple of sets for which $k$ appears in the reduction and let $(X_{k},Y_{k})$ be one of the following pairs:

$$\begin{aligned} & (B\backslash\{k+1,k+4,z_{1}\},A\backslash\{k,k+4,z_{1}\}),\\ & (B\backslash\{k,k+5,z_{2}\},C\backslash\{k+1,k+5,z_{2}\}),\\ & (A\backslash\{k+2,k+6,z_{3}\},B\backslash\{k+3,k+6,z_{3}\}),\\ & (C\backslash\{k+3,k+7,z_{4}\},B\backslash\{k+2,k+7,z_{4}\})\end{aligned}$$

with $z_{i}\in\{1,N\}$ chosen such that $X_{k},Y_{k}\in\mathcal{X}$ holds.

We want to enforce $X_{k}\prec Y_{k}$. By definition, $\max(X_{k})=\max(Y_{k})$ and $\min(X_{k})<\min(Y_{k})$ or $\max(X_{k})<\max(Y_{k})$ and $\min(X_{k})=\min(Y_{k})$. Assume, w.l.o.g. $\max(X_{k})=\max(Y_{k})$ and $\min(X_{k})<\min(Y_{k})$ and let $X_{k}=\{x_{1},x_{2},\dots,x_{l}\}$ and $Y_{k}=\{y_{1},y_{2},\dots,y_{m}\}$ be enumerations of $X_{k}$ resp. $Y_{k}$ such that $i<j$ implies $x_{i}<x_{j}$ resp. $y_{i}<y_{j}$. We add

$$\{x_{l}\},\{x_{l-1},x_{l}\},\dots,\{x_{2},\dots,x_{l}\}$$

and $\{x_{1},x_{l}\}$ to $\mathcal{X}$. This forces by dominance

$$\{x_{2},\dots x_{l}\}\prec\dots\prec\{x_{l-1},x_{l}\}\prec\{x_{l}\}$$

and hence by transitivity and strict independence $X_{k}\prec\{x_{1},x_{l}\}$. Analogously, we can enforce $\{x_{1},y_{1},y_{m}\}\prec\{x_{1}\}\cup Y_{k}$ by adding

$$\{x_{1},y_{1}\},\{x_{1},y_{1},y_{2}\},\dots,\{x_{1},y_{1},\dots,y_{m-1}\}$$

as well as $\{x_{1},y_{1},y_{m}\}$ and $\{x_{1}\}\cup Y_{k}$ to $\mathcal{X}$. Finally we add $\{x_{1},y_{1},y_{m}\}$, $\{x_{1}\}$ and $\{x_{1},y_{1}\}$ enforcing $\{x_{1}\}\prec\{x_{1},y_{1}\}$ by dominance and hence $\{x_{1},y_{m}\}\prec\{x_{1},y_{1},y_{m}\}$ by strict independence. Then, we have $X_{k}\prec Y_{k}$ by

$$X_{k}\prec\{x_{1},x_{l}\}\prec\{x_{1},y_{1},x_{l}\}\prec\{x_{1}\}\cup Y_{k}\prec Y_{k}.$$

The process of producing a positive instance of betweenness from a positive instance of the Dominance Strict Independence problem is the same as for the Maximal Dominance Strict Independence case. However, in order to construct a total order on $\mathcal{X}$, we have to do a bit more. We take the same steps as in the Maximal Dominance Strict Independence case (including the closure under maximal dominance) but additionally, for $A,B\in\mathcal{X}$ with $\max(A)=\max(B)$, $\min(A)=\min(B)$ and $|A|,|B|\leq 3$ we set $A\prec B$ if

1. $$\min(A)=1$$ and $$|A|=2$$ and $$|B|=3$$,
2. $$\min(A)=N$$ and $$|A|=3$$ and $$|B|=2$$,
3. $$|A|=|B|=3$$ and $$A\backslash B<B\backslash A$$.

This order, together with a positive instance of betweenness, maximal dominance and (reverse) strict independence is circle free and decides all possible applications of strict independence. Therefore, we can construct a total order on $\mathcal{X}$ satisfying strict independence and dominance.□

#### The Maximal Dominance Independence Problem

##### *Proof *

We have to adapt the reduction for the Maximal Dominance Strict Independence problem above in two places. We have to change the way we enforce the strict preferences in Figure 1 and Figure 2 and we have to make sure that the order restricted to the sets $V_{1},V_{2},\dots,V_{n}$ is strict.

To enforce, without strict independence, a strict preference between two sets that is not forced by maximal dominance we define for every pair $A,B\in\mathcal{X}$ with $\min(B)\leq\min(A)$, $\max(A)\leq\max(B)$ and $2\leq\max(A)-\min(A)$ a family of sets $\mathcal{S}(A,B)$ forcing $A\prec B$. $\mathcal{S}(A,B)$ contains the following sets

$$\begin{aligned} & \{x_{AB}\},\{y_{AB}\},\{x_{AB},z_{AB}\},\{y_{AB},z_{AB}^{*}\}, \\ & A\cup\{z_{AB}\},B\cup\{z_{AB}^{*}\} \end{aligned}$$

where $\min(A)<y_{AB}<x_{AB}<\max(A)$, $\max(B)<z_{AB}$ and $z_{AB}^{*}<\min(B)$ hold.

Then $A\cup\{z_{AB}\}\prec\{x_{AB},z_{AB}\}$ holds by maximal dominance and, therefore, $\{x_{AB}\}\not\prec A$ and hence $A\preceq\{x_{AB}\}$ holds by “reverse” independence^6^ and analogously, $\{y_{AB}\}\preceq B$. Therefore, transitivity implies $A\prec B$ by $A\preceq\{x_{AB}\}\prec\{y_{AB}\}\preceq B$.

**Fig. 5:**
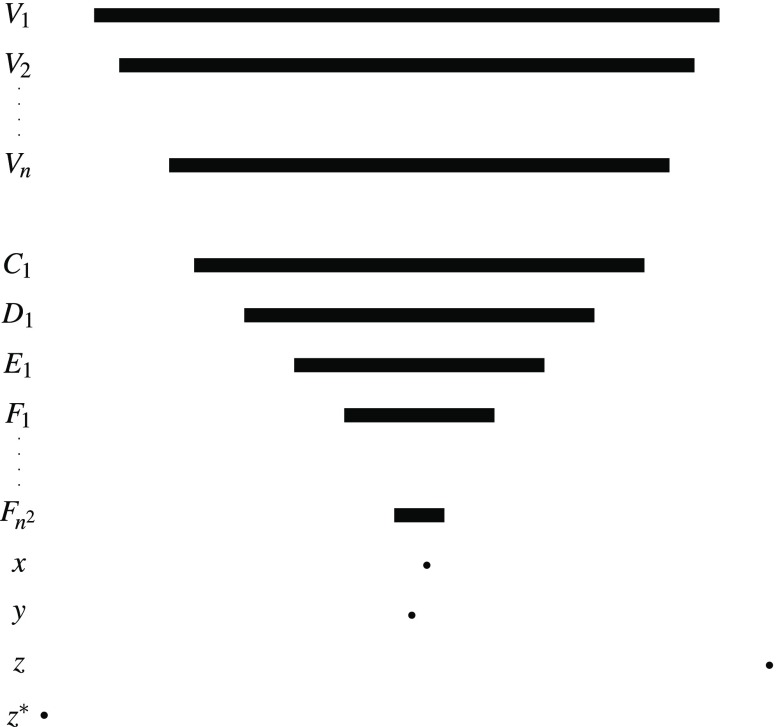
Sketch of the sets $V_{1},\dots,V_{n},C_{1},\dots,F_{1},F_{n^{2}},z$ and $z^{*}$

Using $\mathcal{S}(A,B)$, we can adapt the proof above. We want to construct an instance of the maximal dominance independence problem $(X,<,\mathcal{X})$. We take as $X$ again a set of the form $X=\{1,\dots,N\}$ with the usual linear order, however $N$ has to be larger than in the maximal dominance strict independence case. Namely, we set $N=20n^{3}+28n^{2}+2n+14$. $\mathcal{X}$ contains sets $V_{1},\dots,V_{n}$ similar to the ones in the reductions above (see Figure 5). However, they do not have a common smallest element or common largest element and the smallest element of $V_{1}$ is $4n^{3}+4n^{2}+1$ and largest element $16n^{3}+24n^{2}+2n+14$, i. e.,

$$V_{i}:=\{4n^{3}+4n^{2}+i,\dots,16n^{3}+24n^{2}+2n+14-i\}.$$

We assume, for all families $\mathcal{S}(A,B)$ and $\mathcal{S}(C,D)$ occurring in the reduction, $p_{AB}\neq q_{CD}$ for $p,q\in\{x,y,z\}$ and $(A,B)\neq(C,D)$. As well, for every family $\mathcal{S}(A,B)$, assume $5n^{3}+13n^{2}+n+7\leq y_{AB}$ and $x_{AB}\leq 16n^{3}+17n^{2}+n+7$.

For a triple $(a,b,c)$ in the instance of betweenness we add the following sets as in the two previous reductions:

$$\begin{aligned} & A\backslash\{k\},B\backslash\{k\},B\backslash\{k+1\},C\backslash\{k+1\}, \\ & A\backslash\{k+2\},B\backslash\{k+2\},B\backslash\{k+3\},C\backslash\{k+3\}\end{aligned}$$

where we start with $k=4n^{3}+11n^{2}+n+7$. We force the same preference as in the Maximal Dominance Strict Independence case, by adding the following families:

$$\begin{aligned} & \mathcal{S}(A\backslash\{k\},B\backslash\{k+1\}),\mathcal{S}(C\backslash\{k+1\},B\backslash\{k\}),\\ & \mathcal{S}(B\backslash\{k+3\},A\backslash\{k+2\}),\mathcal{S}(B\backslash\{k+2\},C\backslash\{k+3\}).\end{aligned}$$

**Fig. 6:**
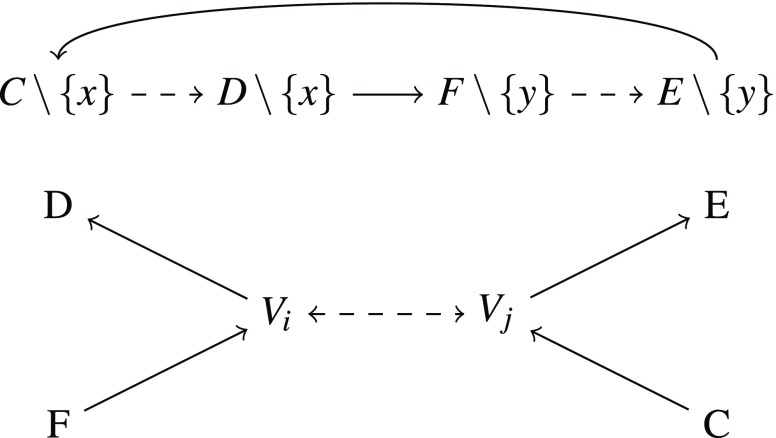
Enforcing strictness without strict independence

We still have to make sure that the order on the sets $V_{1}\dots V_{n}$ is strict. To achieve this we want to use the idea shown in Figure 6, that is to add for every pair $V_{i},V_{j}$ sets that lead to a circle if both $V_{i}\preceq V_{j}$ and $V_{j}\preceq V_{i}$ hold. Let $f(l)=(V_{i},V_{j})$ be an enumeration of all pairs of sets $V_{1},V_{2},\dots,V_{n}$. We add sets $C_{l},D_{l},E_{l}$ and $F_{l}$ that are contained in the “middle parts” of all sets $V_{i}$ such that $C_{l}\subset F_{l^{\prime}}$ holds for all $l^{\prime}<l$. Moreover, we want the following:

$$\begin{aligned} F_{l}& =E_{l}\backslash\{\max(E_{l}),\text{max}^{\prime}(E_{l}),\text{min}^{\prime}(E_{l}),\min(E_{l})\},\\ E_{l}& =D_{l}\backslash\{\max(D_{l}),\text{max}^{\prime}(D_{l}),\text{min}^{\prime}(D_{l}),\min(D_{l})\},\\ D_{l}& =C_{l}\backslash\{\max(C_{l}),\text{max}^{\prime}(C_{l}),\text{min}^{\prime}(C_{l}),\min(C_{l})\}\end{aligned}$$

where $\max^{\prime}(X)$ denotes the second largest element of $X$ and $\min^{\prime}(X)$ the second smallest. We can achieve this by taking for all $l\leq n^{2}$

$$C_{l}:=\{4n^{3}+4n^{2}+n+7l,\dots,16n^{3}+24n^{2}+n+14-7l\}$$

and $D_{l},E_{l}$ and $F_{l}$ accordingly. Furthermore, we add sets $F_{l}\backslash\{\min(F_{l})\}$, $V_{i}\cup\{z_{l}\}$ and $(F_{l}\backslash\{\min(F_{l})\})\cup\{z_{l}\}$ for a unique^7^ $z_{l}<\min(V_{1})$. This ensures $F_{l}\prec F_{l}\backslash\{\min(F_{l})\}\preceq V_{i}$. In a similar fashion we can enforce $V_{i}\prec D_{l}$, $E_{l}\prec V_{j}$ and $V_{j}\prec F_{l}$. Then, we add sets $C_{l}\backslash\{y_{l}\}$, $D_{l}\backslash\{x_{l}\}$, $E_{l}\backslash\{y_{l}\}$ and $F_{l}\backslash\{y_{l}\}$ for $x_{l}=5n^{3}+11n^{2}+n+7+l$ and $y_{l}=5n^{3}+12n^{2}+n+7+l$. Furthermore, we enforce $D_{l}\backslash\{x_{l}\}\prec F_{l}\backslash\{y_{l}\}$ and $E_{l}\backslash\{y_{l}\}\prec C_{l}\backslash\{x_{l}\}$ by adding $\mathcal{S}(D_{l}\backslash\{x_{l}\},F_{l}\backslash\{y_{l}\})$ and $\mathcal{S}(E_{l}\backslash\{y_{l}\},C_{l}\backslash\{x_{l}\})$.

This forces a strict preference between $V_{i}$ and $V_{j}$. Assume otherwise $V_{i}\sim V_{j}$ for a total order satisfying maximal dominance and independence. Then, for $l$ such that $f(l)=(V_{i},V_{j})$ holds, $F_{l}\prec V_{i}\preceq V_{j}\prec E_{l}$ implies $F_{l}\prec E_{l}$. This implies $F_{l}\backslash\{y\}\preceq E_{l}\backslash\{y\}$ because $E_{l}\backslash\{y\}\prec F_{l}\backslash\{y\}$ would imply $E_{l}\preceq F_{l}$, a contradiction. Similarly, $C_{l}\prec V_{j}\preceq V_{i}\prec D_{l}$ implies $C_{l}\backslash\{x\}\preceq D_{l}\backslash\{x\}$. However, then $C_{l}\backslash\{x\}\preceq D_{l}\backslash\{y\}\prec F_{l}\backslash\{y\}\preceq E_{l}\backslash\{y\}\prec C_{l}\backslash\{x\}$ is a circle in $\prec$, contradicting the assumption that $\preceq$ is a total order.

It is straightforward to check that this construction yields a valid reduction analogously to the proof above. The key step is to observe that independence can only be applied to the new sets in cases where it is used in the proof. This is clear for the sets pictured in Figure 6. For the one and two element sets this holds, because the elements are unique and because no three element sets are contained in $\mathcal{X}$.

It remains to check that we can actually pick a unique element every time we want to do this in the reduction. The inner part of $F_{n^{2}}$ has $(16n^{3}+24n^{2}+n+14-7n^{2}-7)-(4n^{3}+4n^{2}+n+7n^{2}+7)=12n^{3}+6n^{2}$ elements. For every triple, we have to pick $12$ unique elements contained in this middle part. These are at most $12n^{3}$ elements. Furthermore, we have to pick for every pair $(V_{i},V_{j})$ $6$, two for $x$ and $y$ and $4$ to enforce preferences, unique elements. There are $n^{2}$ such pairs, so we need $6n^{2}$ elements. Hence we need at most the $12n^{3}+6n^{2}$ elements contained in $F_{n^{2}}$. Furthermore, we need for every pair $(V_{i},V_{j})$ and every triple $4$ elements smaller than $\min(V_{1})$ and the same amount of elements larger than $\max(V_{1})$. This is possible as $\min(V_{1})=4n^{3}+4n^{2}+1$ and $N-\max(V_{1})=(20n^{3}+28n^{2}+2n+14)-(16n^{3}+24n^{2}+2n+14)=4n^{3}+4n^{2}$.□

#### The Dominance Independence problem

##### *Proof *

We construct an instance $(X,<,\mathcal{X})$ of the Dominance Independence problem in a similar fashion as above. We take the same $X$ and $<$ and add the same sets to $\mathcal{X}$ as in the Maximal Dominance Independence case. In order to make the reduction work for the Dominance Independence problem, we have to add more sets.

Observe that maximal dominance is only needed in the reduction for the Maximal Dominance Strict Independence problem to introduce preferences between sets of the form (1) $F_{l}\prec F_{l}\backslash\{\min(F_{l})\}$, (2) $\{x\}\prec\{y\}$, and (3) $R\cup\{z\}\prec S\cup\{z\}$, for sets $R,S$ and $z\in X$. We can enforce these preferences also using independence and regular dominance. (1) is implied by dominance anyway. (2) can be forced by adding $\{x,y\}$ because $\{x\}\prec\{x,y\}\prec\{y\}$ holds by dominance. Finally, we can enforce (3) using the idea from the Dominance Strict Independence reduction.

Now, assume, w.l.o.g., $\min(R)<\min(S)<\max(S)<\max(R)<z$; the other case is similar. Let $R=\{r_{1},\dots,r_{l}\}$ and $S=\{s_{1},\dots,s_{m}\}$ be enumerations of $R$ (resp. $S$) such that $i<j$ implies $r_{i}<r_{j}$ (resp. $s_{i}<s_{j}$). We add $\{z\},\{r_{l},z\},\dots,$ $\{r_{2},\dots,z\},\{r_{1},z\}$ to $\mathcal{X}$. This forces $\{r_{2},\dots,z\}\prec\{z\}$ by dominance and hence by one application of independence $R\cup\{z\}\preceq\{r_{1},z\}$. Analogously, we enforce $\{s_{1},z\}\preceq S\cup\{z\}$ by adding $\{s_{1}\},\{s_{1},s_{2}\},\dots,\{s_{1},\dots,s_{m}\},\{s_{1},z\}$ to $\mathcal{X}$. Finally, we add $\{r_{1}\},\{r_{1},s_{1}\}$ and $\{r_{1},s_{1},z\}$, which leads to $\{r_{1},z\}\preceq\{r_{1},s_{1},z\}$. Then we have $R\cup\{z\}\preceq\{r_{1},z\}\preceq\{r_{1},s_{1},z\}\prec\{s_{1},z\}\preceq S\cup\{z\}$, hence $R\cup\{z\}\prec S\cup\{z\}$.

Checking the correctness of this reduction is straightforward. The correctness proof for the Maximal Dominance Independence case can be adapted to the Dominance Independence case in the same way as the proof of the correctness of the Maximal Dominance Strict Independence case was adapted to the Dominance Strict Independence case.□

## Conclusion

We have shown that the problem of deciding whether a linear order can be lifted to a ranking of sets of objects satisfying a form of dominance and a form of independence is in P or trivial if we do not expect the ranking to be total and NP-complete if we do. In order to prove P‑membership or triviality we constructed such rankings. Rankings of specific sets are useful in several applications, e.g., to eliminate obviously inferior sets of objects from a set of options.

In many applications, the family of sets to be ranked is not given explicitly but implicitly. We expect that a compact representation of the sets increases the computational complexity of decision problems as studied in this paper. As future work we thus want to investigate the complexity blow up caused by a compact representation.

Furthermore, we would like to characterize families that allow an order satisfying (maximal) dominance and (strict) independence. Moreover, it may be possible to find sufficient but not necessary conditions for the existence of such rankings that can be checked in polynomial time. We aim for finding strong forms of such conditions. A related goal is to obtain special classes of families where such a characterization is feasible. A promising candidate are families generated via graphs, where the family is given by the sets of vertices that induce connected subgraphs.

Another item on our agenda is to investigate whether the logic proposed in [8] can be used for specific sets of objects as well. Finally, it would be interesting to study some of the other axioms that have been considered in the literature and see how they behave when one has to rank proper subsets of the whole power set of elements.

## Footnotes

1 In the literature, infinite sets of alternatives are also considered. The results presented in the background section hold also for this case. For the results on computational complexity in the main part, finiteness of the (infinitely many) instances is obviously crucial.

2 Definitions that only differ in the word maximal are combined into one definition using brackets.

3 To be more precise, we show that it is not possible to decide whether such an order exists. However, this also rules out the construction of such an order in polynomial time.

4 This makes sure that we don’t accidentally force a preference between $A\backslash\{k\}$ and $B\backslash\{k\}$.

5 Every total order satisfying strict independence has to satisfy reverse strict independence: If $A\prec B$ holds, then $B\backslash\{x\}\prec A\backslash\{x\}$ would imply $B\prec A$, by strict independence contradicting the irreflexivity of $\prec$, hence by the totality of $\prec$ we have $A\backslash\{x\}\prec B\backslash\{x\}$.

6 Every total order that satisfies independence also satisfies “reverse” independence, i. e., $A\prec B$ implies $A\backslash\{x\}\preceq B\backslash\{x\}$. Otherwise, we would get $B\backslash\{x\}\prec A\backslash\{x\}$ by the totality of $\prec$, hence $B\preceq A$ by independence, contradicting the definition of $\prec$.

7 I. e $z_{l}\neq z_{l^{\prime}}$ for $l\neq l^{\prime}$ and $z_{l}\not\in X$ for all $X\in\mathcal{S}(A,B)$ occurring in the reduction.
